# Alpha-Actinin Is a New Type of House Dust Mite Allergen

**DOI:** 10.1371/journal.pone.0081377

**Published:** 2013-12-06

**Authors:** Su An, Chuanbing Shen, Xiaoyu Liu, Lingling Chen, Xuemei Xu, Mingqiang Rong, Zhigang Liu, Ren Lai

**Affiliations:** 1 Key Laboratory of Animal Models and Human Disease Mechanisms of Chinese Academy of Sciences & Yunnan Province, Kunming Institute of Zoology, Kunming, Yunnan, China; 2 Institute of Allergy and Immunology, Shenzhen University, Shenzhen, China; 3 Clinical Laboratory, The First Affiliated Hospital of Kunming Medical College, Kunming, Yunnan, China; 4 Graduate School of the Chinese Academy of Sciences, Beijing, China; McGill University, Canada

## Abstract

Main indoor allergens for humans are from house dust mites. There are more than 30 allergens in *Dermatophagoides farinae* but only fourteen allergens have been identified from this mite including Der f 1–3, 6, 7, 10, 11, 13–18, and 22. A native allergen protein (Der f 24, 90 kDa) was purified from *D. farinae* by gel filtration and anionic exchange liquid chromatography combined with IgE immunodetection. Its primary structure was determined by Edman degradation, mass spectrometry analysis and cDNA cloning. Enzyme-linked immunosorbent assay inhibition tests (ELISA-IT), immunoblots, basophil activation test (BAT) and skin prick test (SPT) were performed to evaluate the allergenicity. It was identified as an alpha (α)-actinin containing a CaM-like domain with EF-hand motifs. Der f 24 reacted to sera from 85.4% (35/41) of patients on western blot analysis. It reduced ∼20% sera IgE reactivity to *D. farinae* extracts on a competitive ELISA. Eighty percent (8/10) of patients with *D. farinae* allergy showed positive reactions to Der f 24 in skin prick test. The expression of CD63 on basophils from patients was up-regulated by Der f 24 by ∼5.4-fold. Alpha-actinin was identified as a new type of house dust mite allergen. To the best of our knowledge, this is the first report of α-actinin as an allergen.

## Introduction

For humans, house dust mites (HDMs) are major sources of indoor allergens which induce asthma, rhinitis, dermatitis, and other allergic diseases [1], [2]. Many works have been conducted to understand the biological, chemical and structural properties of allergens from HDMs. Allergens from dust mites *Dermatophagoides pteronyssinus* and *D. farinae* (Acari: Pyroglyphidae) have been intensively studied. 23 groups of allergens from dust mite have been listed in the (IUIS) nomenclature dataset, and 21 of them have been identified from *Dermatophagoides* spp (http://www.allergen.org/). Dust mite allergens show significant diversity. Two groups of mite allergens including group 1 (a 25 kDa cysteine protease) and group 2 (a 14 kDa epididymal protein) have been intensively studied. More than 80% of humans with HDM allergy mount an IgE response to the group 1 and more than 90% to the group 2 [3]–[6].

About 20% of patients with HDM allergy have no IgE response to the group 1 and 2 allergens [3]. They had IgE responses to other HDM allergens, which are present in low and variable concentrations in mite extracts (minor allergens). Most of minor allergens’ concentrations in HDM are less than 1% of the group 1 and 2 allergens [3]. Although minor allergens present in low amount in HDM, they can induce high titers of IgE, suggesting that they are potent at low concentration. Previous work has indicated that more than 30 allergens are in the extracts of *D. farinae* although only 14 *D. farinae* allergens have been named [7]. Most of them are in the molecular weight range of 14 to 60 kDa. Especially, few allergens with high molecular weights were found from dust mites although multiple mite proteins have showed interaction with serum IgE of patients [7]. Many investigations with novel allergen identification are still in progress or are yet to be undertaken. In this work, a novel type of allergen (Der f 24) has been identified and characterized from the mite extracts of *D. farina.* Der f 24 belongs to the family of α-actinin, which is never reported as an allergen.

## Material and Methods

### Patient Selection and Skin Prick Test (SPT)

Sera were collected from 41 patients with allergy response to *D. farina*. They have positive SPT responses and the positive responses were further confirmed by measuring *D. farinae*-specific IgE antibodies with the CAP System (Pharmacia & Upjohn Diagnostics AB, Uppsala, Sweden). Of these patients, 15 subjects (42%) are children (10 to 18 years of old, mean 14.3 years) and 21 subjects (58%) are adults (20 to 55 years of old, mean 41.6 years). The characteristics of patients are listed in Table S1. In addition, sera from 16 healthy individuals with a negative SPT response to crude *D. farinae* extract were used as negative controls. SPTs with *D. farinae* extract or purified allergen were performed by using the single-prick technique according to the method described by Dreborg [8]. Sodium chloride (0.9%) and histamine (5 mg/ml) were used as negative and positive control, respectively. Skin response was observed at 15 min and defined as positive when the appearance of a wheal was 3 mm larger than the negative control.

Approval to conduct these studies was obtained from the ethics committee of the Institutional Review Board of the Kunming Institute of Zoology, Chinese Academy of Sciences. All participants were provided written informed consent for the use of blood samples and skin test before study entry. As far as the 15 children’s participation in these studies is concerned, we obtained the written informed consent from their parents and approval of these children to draw about 6 ml venous blood. No children were asked to join the SPTs.

### *D. farinae* Culture and *D. farinae* Extract Preparation

*D. farinae* dust mites were reared at 25°C with a relative humidity of 80% as described by Sasa et al [9]. The main components of the culture medium are yeast extract and mouse diet. Mites were isolated from the medium by using heat-escape method. Totally, 8.0 grams of dust mites were collected, homogenized in 0.05 M Tris-HCl, pH 8.0 and centrifuged at 10,000 rpm for 30 min at refrigerated condition. The supernatant was termed dust mite extract (DME) and lyophilized.

### Allergens Purification from DME

**Figure 1:**
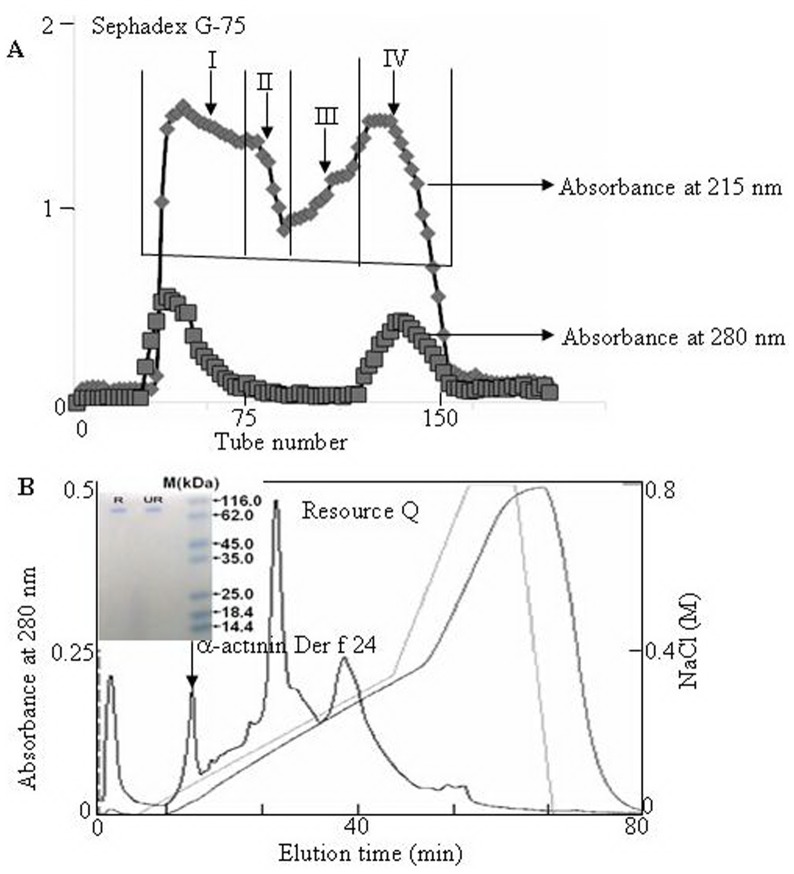
Allergen purification from DME. **A:** 380-75 (Superfine, Amersham Biosciences; 26×100 cm), the absorbance of eluted fractions were monitor at 280 nm. **B:** Pooled fraction I was dialyzed against 20 mM Tris-HCl, pH 8.0 and subjected to further purification by Resource Q anionic exchange column equilibrated with 0.02 M Tris-HCl, pH 8.0 on an AKTA system, and eluted at a flow rate of 1 ml/min with the indicated NaCl gradient in 0.02 M Tris-HCl pH 8.0. *Inserts*: Purified proteins were subjected to SDS-PAGE analysis on a 15% gel. R, reduced; UR, unreduced.

The lyophilized DME sample was dissolved in 100 ml of 0.05 M Tris-HCl, pH 8.0. Aliquot of 10 ml was loaded on a Sephadex G-75 (Superfine, Amersham Biosciences; 26×100 cm) gel filtration column, which was equilibrated with 0.05 M Tris-HCl, pH 8.0. The elution was performed with the same buffer at a flow rate of 0.3 ml/min, with fractions collected of 3.0 ml. The eluted fractions were monitored at 280 nm and assayed for the allergenicity as described below. Fractions containing allergenicity were pooled and further purified by Resource Q anionic exchange column (1 ml volume, GE Healthcare) on an AKTA explorer system (GE Healthcare) as illustrated in Fig. 1.

### Two-dimensional Electrophoresis and Immunoblotting

Fraction I from Sephadex G-75 gel filtration was further separated by two- dimensional electrophoresis. Briefly, fraction I was pooled and treated with 2-D clean-up kit (GE healthcare bioscience) following manufacturer’s instructions. About 50 µg sample was dissolved in 125 µl rehydration buffer (8 M Urea, 0.5% w/v CHAPS, 0.2% w/v DTT, 0.2% v/v IPG pH 3–10, 0.002% w/v bromophenol blue) and loaded onto 7 cm of immobilized pH gradient (IPG) strip pH 3–10NL (GE healthcare bioscience) focusing for 3 hours, having a total of 6 kilovolt-hour (kvh). After isoelectric focusing (IEF), the IPG strips were washed in two stages with equilibration solution containing 6 M urea, 30% v/v glycerol, 2% w/v SDS, 0.002% w/v bromophenol blue and 75 mM Tris-HCL pH 8.8. IPG strips were first equilibrated with 10 mg/ml dithiotheritol (DTT) in equilibration buffer for 15 min and further incubated in the same buffer for another 15 min replacing DTT by 4 mg/ml iodoacetamide. After equilibration, the IPG strips were applied onto the top of 12% SDS-PAGE gels and sealed with 0.5% w/v agarose. SDS-PAGE were run at 5 mA/gel for 15 min as initial migration and increased to 20 mA/gel at 10°C until bromophenol blue dye reached the bottom of each gel. Gels were either stained with coomassie brilliant blue or gels containing proteins with molecular weight around 90 kDa were cut and transferred to polyvinylidene difluoride (PVDF) membrane (Millipore) for IgE-immunoblotting analysis.

Proteins with molecular weight around 90 kDa were electrotransferred to PVDF membranes. Membranes were blocked with 5% non-fat dry milk for 2 hours at room temperature and incubated overnight at 4°C with the pooled serum from dust mites allergic patients diluted at a ration of 1∶20 v/v, followed by 1∶2500 diluted peroxidase-labeled goat anti-human IgE (Kpl InC., Gaithersburg, MD, USA). IgE-binding proteins were detected by using the enhanced chemiluminescence ECL reaction (Amersham) and exposed to Kodak Imaging film. The serum from healthy subjects with no history of dust mites allergy (detected by Pharmacia Unicap 100 system) was used as a negative control.

### SDS-polyacrylamide Gel Electrophoresis (SDS-PAGE) and Protein Quantification

SDS-PAGE was performed by using a Bio-Rad Mini Protean II apparatus under reduced and/or unreduced conditions. Protein sample was boiled for 5 min at the presence of 5× loading buffer before application to polyacrylamide gels (15%). After the electrophoresis, protein bands were observed using a standard Coomassie Blue R250 stain. The protein concentration was determined by a protein assay kit (Bio-Rad, Hercules, CA, USA) with Bovine serum albumin (BSA) as a standard.

### Structural Analysis

The N terminal peptide and partial interior peptide fragments produced by trypsin digestion were undertaken by automated Edman degradation on an Applied Biosystems pulsed liquid-phase sequencer, model 491 (Applied Biosystems, CA, USA). The mass spectra were obtained by using a nano-electrospray quadripole time of flight (ESI-QUAD-TOF) mass spectrometer (QTOF II, Micromass, Manchester, UK). The instrument was operated in a positive ion mode under the following parameters: capillary voltage, 3.0 Kv; nebuliser gas (N_2_) flow rate, 80 L/h; desolvation gas (N_2_) flow rate, 400 L/h. A range of different collision energies were used depending on the m/z value of the selected precursor to produce optimal fragmentation. The mass spectra data were analyzed by using MASCOT software (London, UK; www.matrixscience.com) and searched against several publicly available databases (NCBI nr, www.ncbi.nlm.nih.gov; Swissprot, www.ebi.ac.uk/swissprot/).

### SMART cDNA Synthesis and cDNA Library Construction

TRIzol Reagent (Invitrogen) was used to extract total RNA from 80 mg *D. farinae* dust mites. mRNA was purified by an Oligotex mRNA Mini kit (Qiagen) according to manufacturer’s instruction. Creator™ SMART™ cDNA Library Construction Kit (Clontech, Palo Alto, CA) was used for cDNA synthesis by SMART™ techniques. The first strand of cDNA was synthesized by using two primers including 3 SMART CDS III/3′ PCR primer (5′-ATTCTAG AGGCCGAGGCGGCCGACATG-d(T)_30_N__1_N-3′ (N = A, G, C, or T; N__1_ =  A, G, or C)) and SMART IV oligonucleotide (5′-AAGCAGTGGTATCAAC GCAGAGTGGCCATTACGGCCGGG-3′) and SMART MMLV reverse transcriptase. The second strand was synthesized by using Advantage polymerase, 5′ PCR primer (5′-AAGCAGTGGTATCAACGCAGAGT-3′) and CDS III/3′ PCR primer (5′-ATTCTAGAGGCCGAGGCGGCCGACATG-3′). Using the cDNA product, a directional cDNA library was constructed with a plasmid cloning kit (SuperScriptTM plasmid System; Invitrogen) following manufacturer’s instructions, producing a library of about 2.5×10^5^ independent colonies.

### Screening of cDNA Library

The specific primer (5′-GC(A/T/CG) GC(A/T/CG)CC(A/T/CG)TA(T/C)AA(T/C) AA(T/C)TGGTT-3′), AAPFNNWLDGAR which was designed according to the peptide sequences determined by Edman degradation or ESI-QUAD-TOF mass spectrometry analysis, and 3′ PCR primer CDS III as mentioned above were used for the screening of cDNA library. The PCRs were performed using Advantage polymerase from Clontech as follows: 2 min at 94°C followed by 30 cycles of 10 s at 92°C, 30 s at 50°C, and 40 s at 72°C. The PCR products were recovered by DNA Gel Extraction Kit (Tiangen China) and ligated into pMD19-T vector (TaKaRa Biotechnology Dalian Co., Ltd., China) following manufacturer’s instructions. DNA sequencing was performed on an Applied Biosystems DNA sequencer, model ABI PRISM377.

### Immunoblotting Analysis with Sera from Dust Mite Allergic Patients

The purified allergen was subjected to SDS-PAGE on a 15% gel. Protein band on the gel was electro-transferred to nitrocellulose membrane (Bio-Rad, Hercules, CA, USA) for immunoblotting analysis. Membrane was blocked for 2 hours with 5% BSA in room temperature. The membrane was incubated with patients’ sera 1∶10 (v/v) diluted in 5% BSA-PBST (PBST: PBS containing 0.05% Tween 20) overnight at 4°C under constant agitation on a rotary shaker. After washing three times with PBST, the membrane was incubated with horseradish peroxidase (HRP)-labeled goat anti-human IgE monoclonal antibody (1∶4000, KPL Inc., Gaithersburg, MD, USA) in 5% BSA-PBST. After washing with PBST for three times, fluorescence was visualized by incubating the membrane with HRP substrate.

### Enzyme-linked Immunosorbent Assay (ELISA) and ELISA Inhibition

Sera IgE antibodies specific for purified allergen was measured by indirect ELISA as described [10], [11]. Briefly, 2 µg purified allergen in 100 µl carborate-biocarborate buffer (15 mM Na_2_CO_3_ and 35 mM NaHCO_3_, pH 9.6) was incubated in a 96-well microtiter plate (Nunc, Roskilde. Denmark) overnight at 4°C. The plate was blocked for 30 min at 37°C with 200 µl 3% BSA in PBS, and then the plate was incubated with 50 µl 1∶10 (v/v) diluted serum in 0.1% BSA-PBST for 40 min at 37°C. After IgE binding, peroxidase-labeled goat anti-human IgE (Kpl InC., Gaithersburg, MD, USA,1∶2000) was added into the plate for 30 min incubation at 37°C. Each incubating step was followed by washing 3 times with PBST. The color was developed by adding 100 µl chromogenic substrate to each well and stopped by the addition of 50 µl 2 M sulphuric acid. The absorbance at 450 nm was measured by a microplate reader at 450 nm (Epoch Etock, BioTek). Two patients (6 and 8) whose sera recognized the purified allergen was used for ELISA inhibition assay according to previously method [11]. Patients sera (diluted 1∶20 in 2% BSA, 0.05% Tween 20 PBS) were pre-incubated with purified allergen or DME (final concentration: 0.0003–30 µg/ml) at 37°C for 1 hour and then were added to the microtiter plate previously coated with 100 µl per well of 20 µg/ml DME. The subsequent steps were the same as those for direct ELISA as described above. All ELISAs were tested in triplicate and the data were presented as the mean ± SD.

### Basophil Activation Test (BAT)

Allergens can activate basophils by inducing the up-regulation of CD63 on the basophil surface, which was considered as the marker of basophil activation [12], [13]. Venous blood was collected from 6 dust-mite-allergic patients and 6 non-allergic subjects. Peripheral blood mononuclear cells (PBMCs) were isolated by using Ficoll-Paque density gradient and resuspended in stimulation buffer (20 mM HEPES,133 mM NaCl, 7 mM KCl, 3.5 mM MgCl_2_, 1 mg/ml BSA, 2 ng/ml IL-3, pH 7.4). Then 3 aliquots of PBMCs from a dust-mite-allergic patient or a non-allergic subject were incubated with 1.0 µg/ml testing allergen, goat anti-human IgE antibody (Kpl InC., Gaithersburg, MD, USA) and stimulation buffer at 37°C for 40 min, respectively. The goat anti-human IgE antibody was taken as positive control and the stimulation buffer was used to evaluate the basal CD63 level. The reaction was stopped on ice and the basophils were incubated with anti-human CD63-FITC antibody and anti-human CD193 (CCR3)-PE antibody (Biolegend, CA, USA) at 4°C for 15 min. The CCR3 and low granularity were used for gating basophils [13]. Basophil activation was quantified as the percentage of CD63-positive basophils. Basophil surface markers were analyzed at 488 nm on a FACScan flow cytometer (Becton Dickinson, Franklin Lakes, NJ, USA) using FACSDiva™ software.

### Statistical Analysis

Statistical significance was analyzed by using the SPSS 13.0 version for *t*-test. Data were shown as mean ± SD (Standard deviation) and the data for direct ELISA were plotted in a scatter plot. Mann-Whitney *U*-test was used to analyze the nonparametric data. *P*-value of less than 0.05 was taken as statistically significant.

## Results

### Allergen Purification from *D. farinae* Mite Extracts (DME)

The supernatant of DME was divided into four fractions by Sephadex G-75 gel filtration as illustrated in Fig. 1A. It was found that there are allergenic proteins in fraction I. Fraction I was subjected to further purification by Resource Q anionic exchange column on an AKTA system (Fig. 1B). An allergen named Der f 24 was purified. SDS-PAGE analysis indicated that it was a homogenized protein (*inserts* in Fig. 1B). Der f 24 was determined as an α-actinin as described below.

### Analysis of Dust Mite Allergens with Molecular Weight around 90 kDa by 2-DE and IgE Immunoblotting

**Figure 2:**
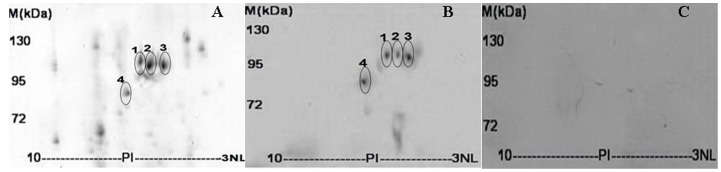
The identification of *D. farinae* allergens with molecular weight around 90 kDa by coupling 2-DE with 2-D immunoblotting. Fraction I from Sephadex G-75 gel filtration was further separated by 2-DE and stained with Coomassie G-250 (A) or transferred to PVDF membranes followed by IgE immunoblotting with dust mite allergic (B) and healthy human sera (C), respectively.

As mentioned above, fraction I was further separated by 2-DE and the IgE-binding ability of its components was detected by IgE immunoblotting as illustrated in Fig. 2. There were four IgE immune-reactive spots matched well with protein spots in 2-DE of fraction I. As listed in Table S2, these protein spots were found to belong to 2 different protein families by ESI-QUAD-TOF-based mass spectrometry analysis (Fig. S1). Among them, spots 1, 2 and 3 belong to a known allergen (chintinase Der f 15) of *D. farinae*, spot 4 belongs to alpha-actinin families. Der f 15 with 3 spots may represent its different isoforms.

### Structural Characterization of Der f 24 from the *D. farinae* Dust Mites

**Figure 3:**
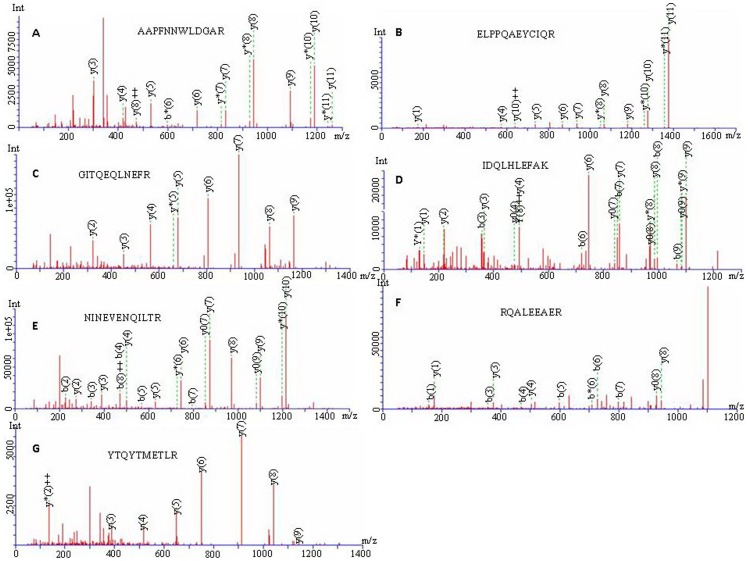
Amino acid sequences of seven interior fragments (A–G) determined by ESI-QUAD-TOF mass spectrometry analysis.

**Figure 4:**
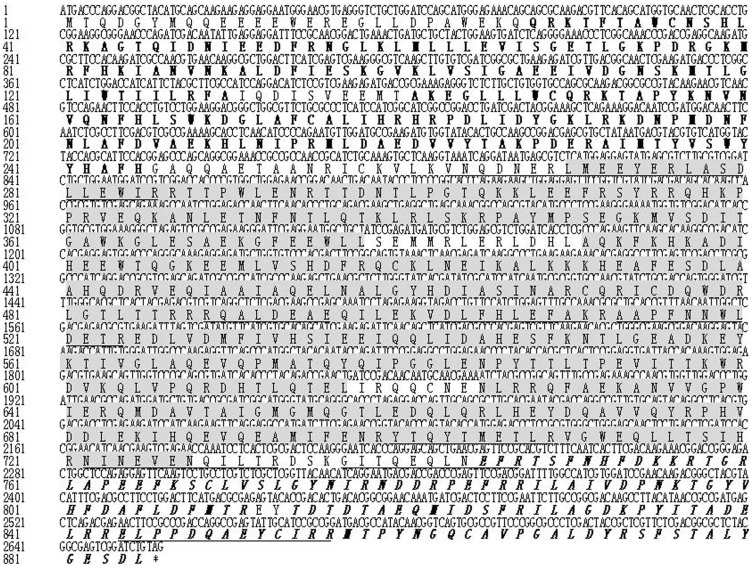
cDNA sequence encoding Der f 24 and the deduced amino acid sequence. The sequences underlined were determined by mass spectrometry analysis and Edman degradation. *: stop codon. Bolded sequences are calponin homology domains (actin-binding domains); Shaded seqeunces are spectrin domains; Bolded and italized squences are EF-hand motifs.

The α-actinin named Der f 24 was purified from DME (Fig. 1). Its molecular mass is ∼90 kDa as determined by SDS-PAGE analysis. Amino acid sequences of several peptide fragments of Der f 24 were obtained by Edman degradation and/or ESI-QUAD-TOF-based mass spectra analysis (Fig. 3). Seven peptide fragments of Der f 24 were sequenced (AAPFNNWLDGAR, ELPPQAEYCIQR, GITQEQLNEFR, IDQLHLEFAK, NINEVENQILTR, RQALEEAER, YTQYTMETLR). Based on the sequences of these peptide fragments, degenerate primers were designed to clone the cDNA sequence encoding the allergen. The cDNA sequence encoding Der f 24 was cloned from the *D. farinae* cDNA library (GenBank accession number KC305498) as illustrated in Fig. 4. The precursor is composed of 885 amino acid residues (aa) (Fig. 4). There are two copies of calponin homology (CH) domain in the N-terminus, three spectrin domains in the middle position, and two EF-hand motifs in the C-terminus (Fig. 4).

### Immunoreactivity to IgE

**Figure 5:**
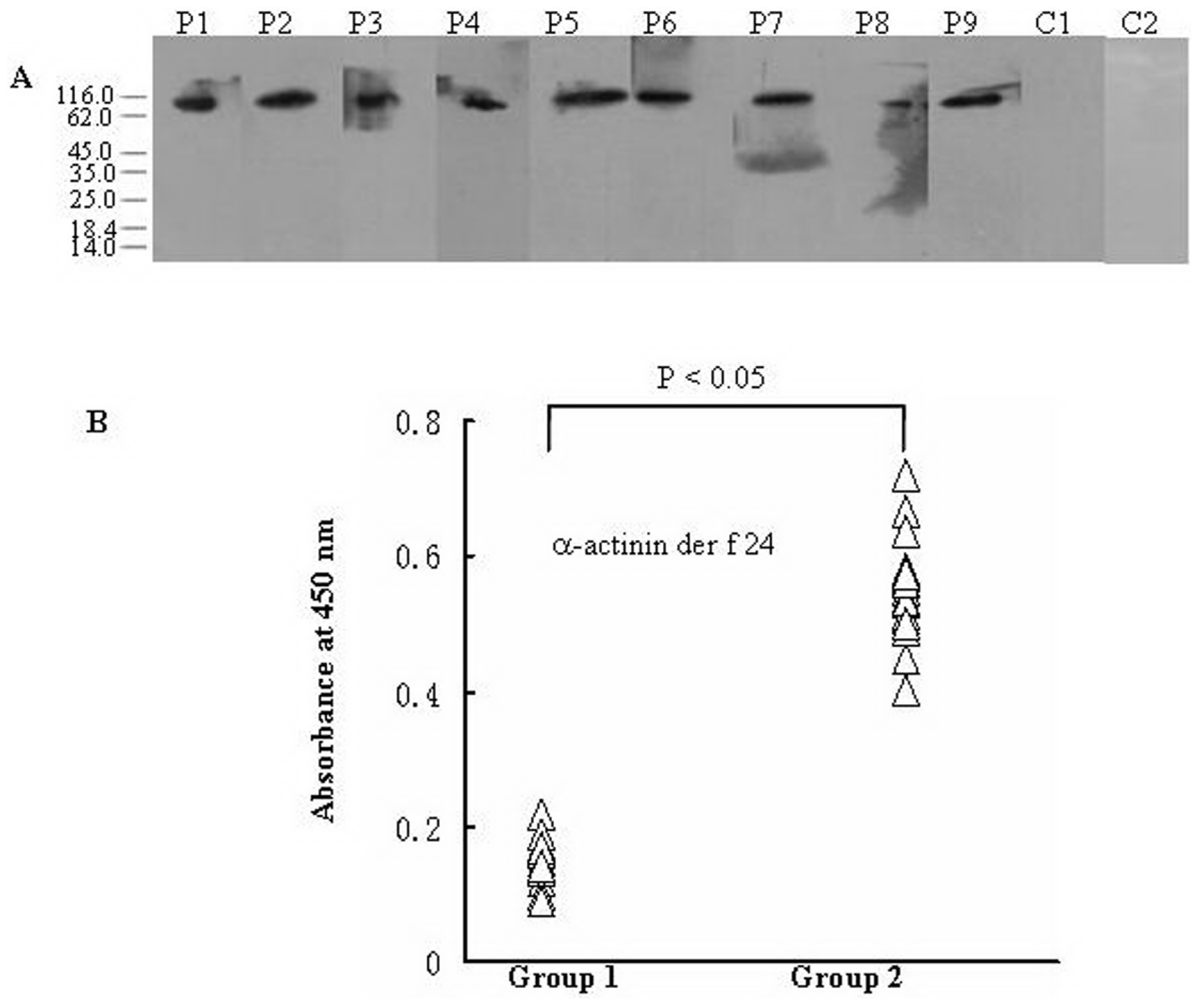
Specific IgE reactivity to allergen Der f 24. A: Immunobloting analysis of specific IgE reactivity to allergen Der f 24 in the sera from the patients with dust mite allergy. Lanes 1–9, representatives of sera from allergic subjects. Lane 10 & 11 marked as C, sera from healthy individuals as negative control. B: Evaluation of specific IgE reactivity to allergen Der f 24 by direct ELISA. Group 1: the sera from healthy control subjects, group 2: the sera from Der f 24-positive patients.

To determine the allergenicity of native Der f 24 protein, the immunoblotting was performed using individual sera from 41 dust mite allergic patients. The result demonstrated that sera IgE from 35 of 41 (85.4%) dust mite allergic patients reacted to Der f 24. IgE-binding ability of Der f 24 in a representative group of 9 patients and two controls is illustrated in Fig. 5A. IgE-binding bands with molecular weight around 90 kDa (Der f 24) were positive for patients, but completely negative in healthy subjects. The specific immunoreactivity of IgE against purified Der f 24 allergen was further confirmed by direct ELISA (Fig. 5B). In comparison with the sera from healthy control subject, the IgE-reactivity of Der f 24 in the sera from positive patients increased by 4.1 folds (Fig. 5B).

**Figure 6:**
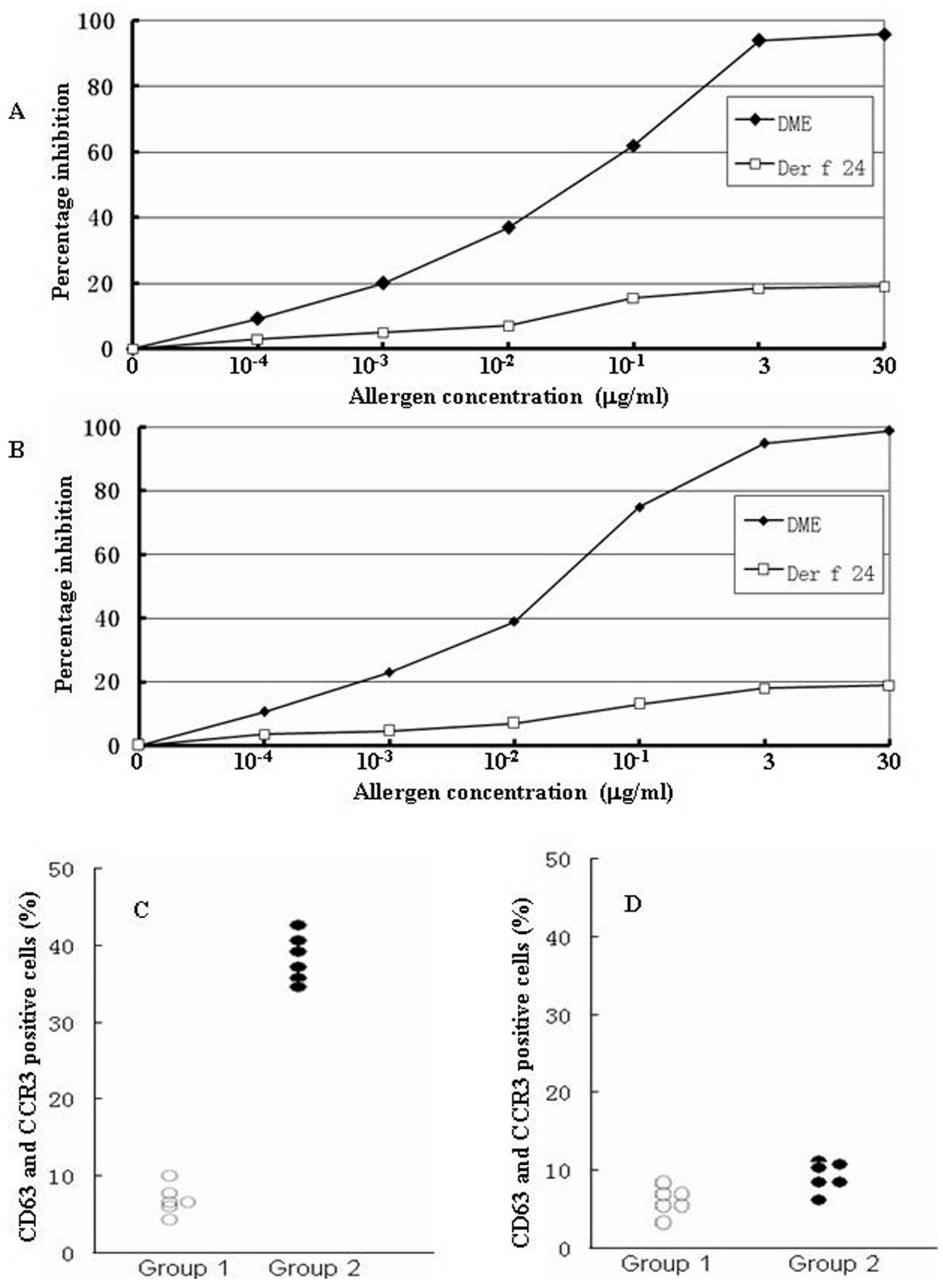
Purified allergens inhibited the patients’ serum IgE binding to the coated DME in a dose-dependent manner (A, B) and induction of basophil activation by purified Der f 24 (C, D). A and B represent the serum from dust mite allergy patients 6 and 8, respectively. Allergen concentrations ranged from 0 to 30 µg/ml. DME: dust mite extracts. Der f 24 was incubated with 6 dust mite allergenic patients’ PBMC (C) or 6 healthy subjects’ PBMC (D). Group 1: using the stimulation buffer to evaluate the basal CD63 level. Group 2: Der f 24.

For ELISA inhibition assay, two patients’ sera (6 and 8) which had positive reactions to Der f 24 were chosen. Different concentrations of Der f 24 or DME were incubated with the serum. They inhibited the patients’ serum IgE binding to the coated DME in a dose-dependent manner (Fig. 6A & B).

### Basophil Activation Analysis

To analyze basophil activation of native Der f 24 allergen, the optimal concentration 1 µg/ml of the allergen was applied to this experiment. In comparison with healthy control, Der f 24 induced approximately up to 5.4 folds increase in CD63 and CCR3 double-positive cells following incubating the allergens with PBMC from patients with dust mite allergy (Fig. 6C & D).

### Skin Prick Testing

The allergic activity of Der f 24 was evaluated by SPT. Eight (80%) of 10 dust mite allergic patients showed positive reaction to this allergen (Table 1).

**Table 1:** Results of skin prick tests.

Subjects	Net wheal size (mm)
	Der f 24
1	<3
2	6.6
3	5.9
4	8.3
5	<3
6	7.5
7	6.1
8	5.4
9	7.5
10	5.8

Patients with mite allergy; Net wheal size in mm = allergen reaction size - negative control size; Negative-control wheal size: mean, 0.41 mm; median, 0 mm; range, 0–3 mm.

## Discussion

A large amount of work has demonstrated that there is extreme diversity of allergens in house dust mites but less than half of these allergens have been identified and characterized (7). Most of allergens from house dust mites are in the molecular weight range of 14 to 60 kDa. In the dust mite *D. farinae*, there is only an identified allergen (chitinase, 95 kDa) with a molecular weight larger than 60 kDa (7). In the work reported by Mao et al, two protein groups from *D. farinae* with a high molecular weight showed significant sera IgE reactivity: one group with molecular weight range of 80 to 83 kDa, and the other group with molecular weight range of 95 to 101 kDa (7). These results suggest that there are multiple allergens with high molecular weights in *D. farinae* mite.

In this work, a novel allergen named Der f 24 with high molecular weight was purified (Fig. 1) and characterized from the dust mite *D. farinae*. It is an α-actinin with a molecular weight of 90 kDa (Fig. 1 & 4). Its primary structure and allergenicity were characterized by Edman degradation, mass spectrometry (Fig. 3), cDNA cloning (Fig. 4), immunoblotting (Fig. 5) competitive ELISA, BAT (Fig. 6), and SPT (Table 1).

As far as we know, no α-actinin allergen has been reported in previous literatures. α-actinin belongs to the protein family of spectrin containing calmodulin (CaM)-like domain in its C-terminus. There are EF-hand motifs in the CaM-like domain. As illustrated in Fig. 4, Der f 24 is composed of two actin-binding domains, three spectrin domains, and two EF-hand domains. It has been reported that many EF-hand–type proteins with a variable number of EF motifs are allergens including tree pollen allergens (Bet v 4, Ole e 3, Ole e 8), grass pollens (Phl p7), rape seed (Bra n 1, Bra n 2), fish allergens (Gad m 1, Sal s 1), frog allergens (Ran e 1, Ran e 2), and the cockroach allergen (troponin C, Bla g 6) [14]–[17]. These results suggest that EF-hand–containing proteins have the ability to induce allergy. Der f 24 from *D. farinae* was identified as an α-actinin containing EF-hand and demonstrated to be an allergen. It is a new type of HDM allergen.

Using western blotting and SPT techniques, we proved that Der f 24 is an important allergen of *D. farinae*. Der f 24 reacted to sera from 85.4% of patients with *D. farinae* allergy (Table 1). However, Der f 24 is not the only allergen in the dust mite, as it could only abolish up to 20% of binding capacity of serum IgE to the coated DME, whereas DME completely blocked the immunological reaction in the competitive ELISA (Fig. 6). The ability of Der f 24 in activating *D. farinae* -allergy-serum-sensitized basophils also indicated that this α-actinin from the dust mite possesses allergen function (Fig. 6).

The development of effective diagnostic and therapeutic approaches depends on the continuity of research of HDM allergens and the identification of allergen diversity [18]–[21]. The current work identified a new type of *D. farinae* dust mite allergen, which belongs to α-actinin with a molecular weight of 90 kDa. It is another group of allergen with high molecular weight from *D. farinae*. The discovery of new type of allergen will be helpful for HDM allergy diagnosis and therapy, especially for patients without response to HDM major allergens. In addition, the discovery of α-actinin allergen may provide more insights to understand the diversity, characterization of allergens.

## Supporting Information

Figure S1 **The peptide mass spectra of 2** ***D. farinae*** **allergens (Der f 15 with 3 isoforms and Der f 24) analyzed by ESI-QUAD-TOF mass spectrometry.**
(DOC)

Table S1 The clinical information of 41 patients in this research.
(DOC)

Table S2 *D. farinae* allergens with molecular weight around 90 kDa identified by 2-DE and ESI-QUAD-TOF mass spectrometry.
(DOCX)

## References

[1] Tovey ER, Chapman MD, Platts-Mills TA (1981) Mite faeces are a major source of house dust mite allergens. Nature 289: 592–593. 7464922 10.1038/289592a0

[2] Hong CS, Lee MK, Oh SH (1991) Identification of major allergens from the house dust mites, *Dermatophagoides farinae* and *Dermatophagoides pteronyssinus*, by electroblotting. Yonsei Med J 32: 24–32. 1877252 10.3349/ymj.1991.32.1.24

[3] Thomas WR, Smith WA, Hales BJ (2004) The allergenic specificities of the house dust mite. Chang Gung Med J 27: 563–569. 15553602

[4] Van Der Veen MJ, Jansen HM, Aalberse RC, Van der Zee JS (2001) Der p 1 and Der p 2 induce less severe late asthmatic responses than native *Dermatophagoides pteronyssinus* extract after a similar early asthmatic response. Clin Exp Allergy 31: 705–714. 11422129 10.1046/j.1365-2222.2001.01120.x

[5] Trombone AP, Tobias KR, Ferriani VP, Schuurman J, Aalberse RC, et al (2002) Use of a chimeric ELISA to investigate immunoglobulin E antibody responses to Der p 1 and Der p 2 in mite-allergic patients with asthma, wheezing and/or rhinitis. Clin Exp Allergy 32: 1323–1328. 12220471 10.1046/j.1365-2745.2002.01455.x

[6] Meyer CH, Bond JF, Chen MC, Kasaian MT (1994) Comparison of the levels of the major allergens Der p I and Der p II in standardised extract of the house dust mite, *Dermatophagoides pteronyssinus* . Clin Exp Allergy 24: 1041–1048. 7874602 10.1111/j.1365-2222.1994.tb02741.x

[7] Le Mao J, Mayer CE, Peltre G, Desvaux FX, David B, et al (1998) Mapping of *Dermatophagoides farinae* mite allergens by two-dimensional immunoblotting. J Allergy Clin Immunol 102: 631–636. 9802372 10.1016/s0091-6749(98)70280-5

[8] Dreborg S, Foucard T (1983) Allergy to apple, carrot and potato in children with birch pollen allergy. Allergy 38: 167–172. 6846743 10.1111/j.1398-9995.1983.tb01602.x

[9] Sasa M, Miyamoto J, Shinohara S, Suzuki H, Katsuhata A (1970) Studies on mass culture and isolation of *Dermatophagoides farinae* and some other mites associated with house dust and stored food. Jpn J Exp Med 40: 367–372. 5313054

[10] Ma D, Li Y, Dong J, An S, Wang Y, et al (2011) Purification and characterization of two new allergens from the salivary glands of the horsefly, *Tabanus yao* . Allergy 66: 101–109. 20608917 10.1111/j.1398-9995.2010.02435.x

[11] An S, Ma D, Wei JF, Yang X, Yang HW, Yang H, et al (2011) A novel allergen Tab y 1 with inhibitory activity of platelet aggregation from salivary glands of horseflies. Allergy 66: 1420–1427. 21848516 10.1111/j.1398-9995.2011.02683.x

[12] Schuerwegh AJ, Ebo DG, Bridts CH, De Clerck LS, Stevens WJ (2001) CD63 expression on basophils of nonallergic controls and patients allergic to wasp. J Allergy Clin Immunol 108: 150–152. 11447407 10.1067/mai.2001.116125

[13] Pruzansky JJ, Grammer LC, Patterson R, Roberts M (1983) Dissociation of IgE from receptors on human basophils. I. Enhanced passive sensitization for histamine release. J Immunol 131: 1949–1953. 6194222

[14] Swoboda I, Bugajska-Schretter A, Verdino P, Keller W, Sperr WR, et al (2002) Recombinant carp parvalbumin, the major cross-reactive fish allergen: a tool for diagnosis and therapy of fish allergy. J Immunol 168: 4576–4584. 11971005 10.4049/jimmunol.168.9.4576

[15] Elsayed S, Bennich H (1975) The primary structure of allergen M from cod. Scand J Immunol 4: 203–208. 1145128 10.1111/j.1365-3083.1975.tb02618.x

[16] Hindley J, Wunschmann S, Satinover SM, Woodfolk JA, Chew FT, et al (2006) Bla g 6: a troponin C allergen from *blattella germanica* with IgE binding calcium dependence. J Allergy Clin Immunol 117: 1389–1395. 16751002 10.1016/j.jaci.2006.02.017

[17] Ayuso R, Grishina G, Ibáñez MD, Blanco C, Carrillo T (2009) Bencharitiwong R, et al. Sarcoplasmic calcium-binding protein is an EF-hand-type protein identified as a new shrimp allergen. J Allergy Clin Immunol 124: 114–120. 19523674 10.1016/j.jaci.2009.04.016

[18] Lin KL, Hsieh KH, Thomas WR, Chiang BL, Chua KY (1994) Characterization of Der p V allergen, cDNA analysis, and IgE mediated reactivity to the recombinant protein. J Allergy Clin Immunol 94: 989–996. 7798547 10.1016/0091-6749(94)90117-1

[19] Shen DH, Chua KY, Lin WL, Hsieh KH, Thomas WR (1995) Characterization of the house dust mite allergen Der p 7 by monoclonal antibodies. Clin Exp Allergy 25: 416–422. 7553244 10.1111/j.1365-2222.1995.tb01072.x

[20] Aki T, Kodama T, Fujikawa A, Miura K, Shigeta S, et al (1995) Immunochemical characterization of recombinant and native tropomyosins as a new allergen from the house dust mite, *Dermatophagoides farinae* . J Allergy Clin Immunol 96: 74–83. 7622766 10.1016/s0091-6749(95)70035-8

[21] Hales BJ, Martin AC, Pearce LJ, Laing IA, Hayden CM, et al (2006) IgE and IgG anti-house dust mite specificities in allergic disease. J Allergy Clin Immunol 118: 361–367. 16890759 10.1016/j.jaci.2006.04.001

