# Supplementary material for: Alpha-Actinin Is a New Type of House Dust Mite Allergen
Source: PLoS One. 2013 Dec 6;8(12):e81377. doi: 10.1371/journal.pone.0081377 (PMC3855699; doi:10.1371/journal.pone.0081377)
Supplement: Figure S1 — The peptide mass spectra of 2 D. farinae allergens (Der f 15 with 3 isoforms and Der f 24) analyzed by ESI-QUAD-TOF mass spectrometry. (DOC) [file pone.0081377.s001.doc]

**Figure S1. The peptide mass spectra of 2 *D. farinae* allergens (Der f 15 with 3 isoforms and Der f 24) analyzed by ESI-QUAD-TOF mass spectrometry.**

**Fig. S1A-1**

**
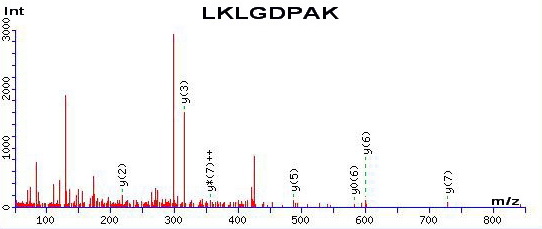
**

**Fig. S1A-2**

**
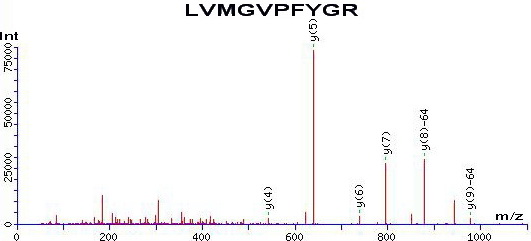
**

**Fig. S1A-3**

**
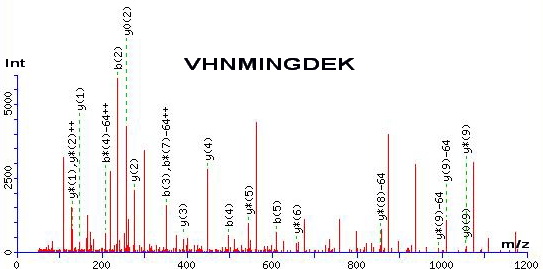
**

**Fig. S1A-4**

**
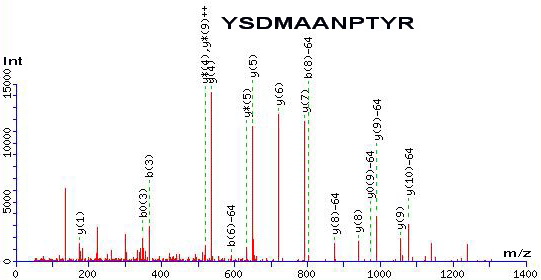
**

**Fig. S1A-5**

**
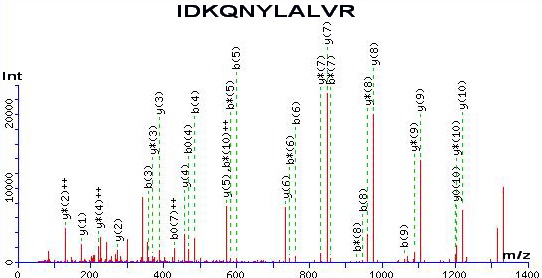
**

**Fig. S1A-6**

**
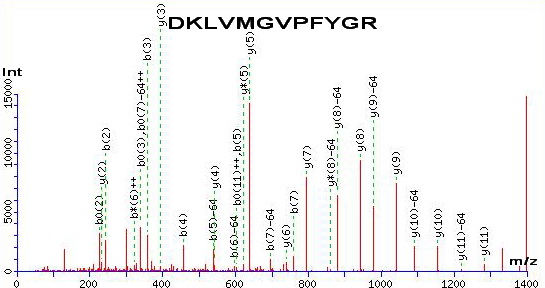
**

**Fig. S1A The mass spectra of peptide fragments from allergen Der f 15 (Fig. 2A &**

**B, spot 1) analysed by ESI-QUAD-TOF mass spectrometry.**

**Fig. S1B-1**

**
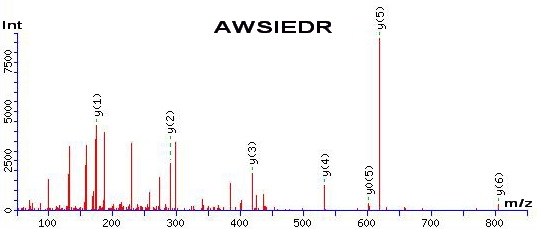
**

**Fig. S1B-2**

**
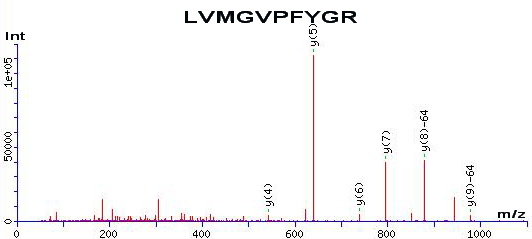
**

**Fig. S1B-3**

**
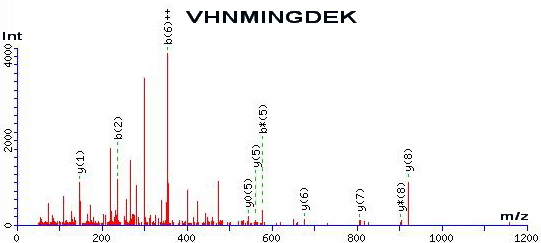
**

**Fig. S1B-4**

**
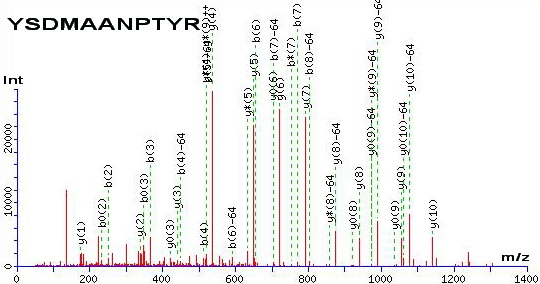
**

**Fig. S1B-5**

**
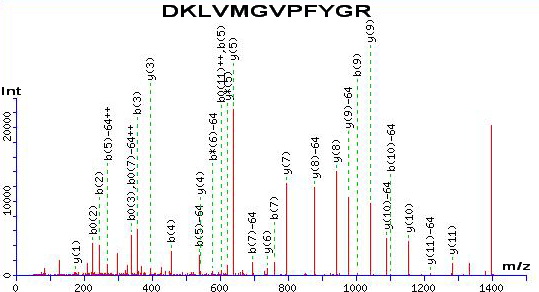
**

**Fig. S1B-6**

**
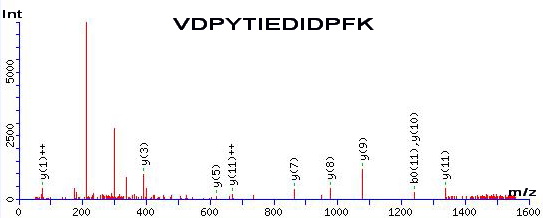
**

**Fig. S1B The mass spectra of peptide fragments from allergen Der f 15 (Fig. 2A &**

**B, spot 2) analysed by ESI-QUAD-TOF mass spectrometry.**

**Fig. S1C-1**

**
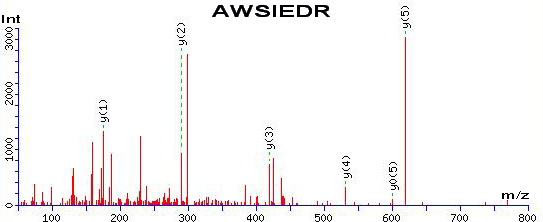
**

**Fig. S1C-2**

**
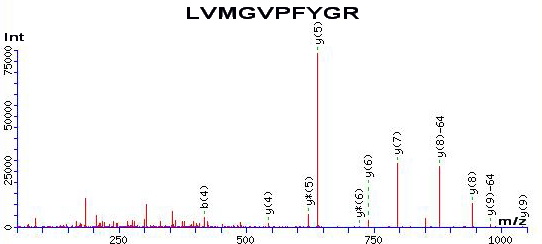
**

**Fig. S1C-3**

**
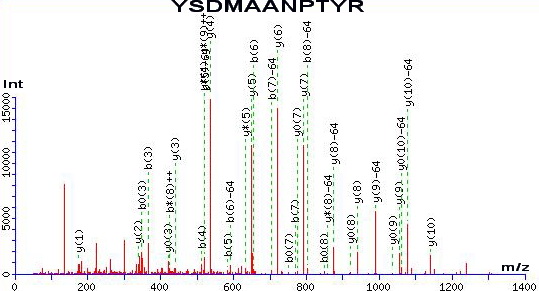
**

**Fig. S1C-4**

**
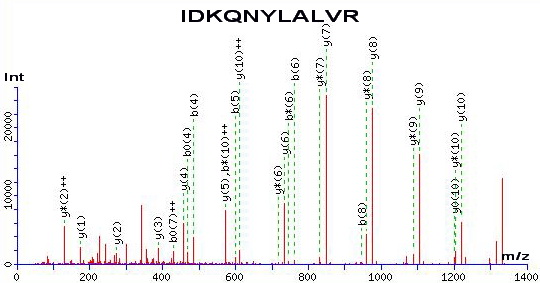
**

**Fig. S1C-5**

**
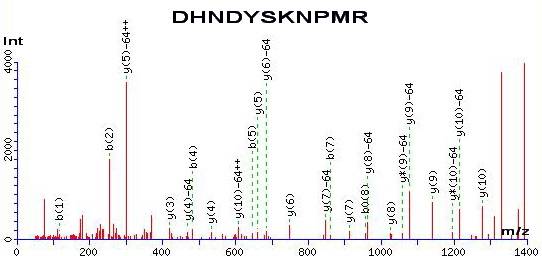
**

**Fig. S1C-6**

**
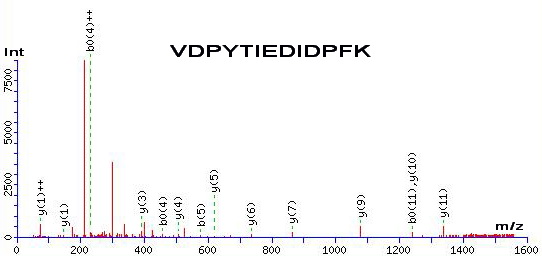
**

**Fig. S1C The mass spectra of peptide fragments from allergen Der f 15 (Fig. 2A &**

**B, spot 3) analysed by ESI-QUAD-TOF mass spectrometry.**

**Fig. S1D-1**

**
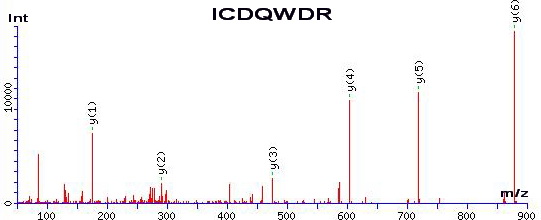
**

**Fig. S1D-2**

**
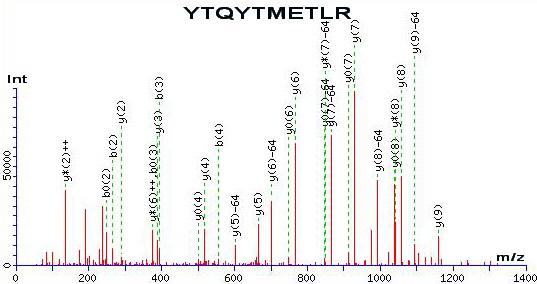
**

**Fig. S1D-3**

**
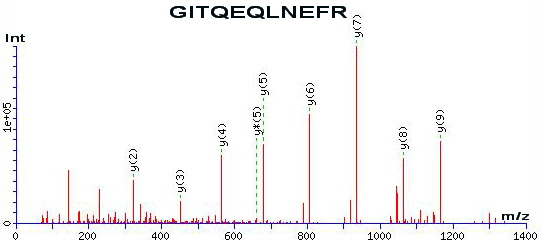
**

**Fig. S1D-4**

**
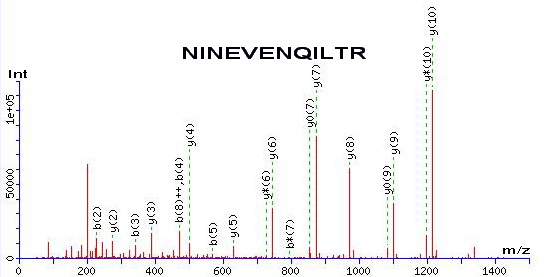
**

**Fig. S1D-5**

**
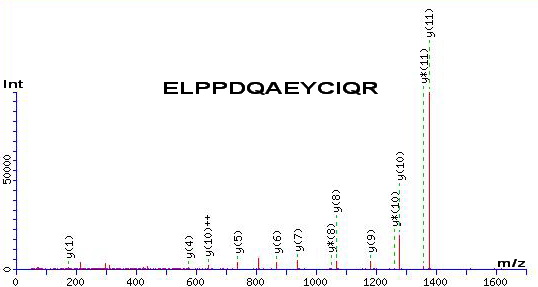
**

**Fig. S1D The mass spectra of peptide fragments from allergen alpha-actinin Der f**

**24 (Fig. 2A &B, spot 4) analysed by ESI-QUAD-TOF mass spectrometry.**
