# Supplementary material for: Alpha-Actinin Is a New Type of House Dust Mite Allergen
Source: PLoS One. 2013 Dec 6;8(12):e81377. doi: 10.1371/journal.pone.0081377 (PMC3855699; doi:10.1371/journal.pone.0081377)
Supplement: Table S1 — The clinical information of 41 patients in this research. (DOC) [file pone.0081377.s002.doc]

Table S1 The clinical information of 41 patients in this research.


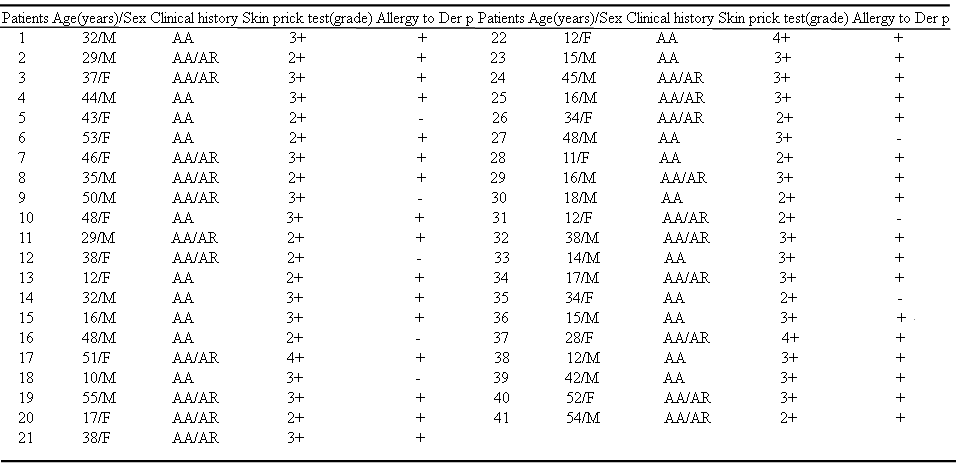


AA: Allergic asthma; AR: Allergic rhinitis; Der p: *Dermatophagoides* *pteronyssinus*; Skin prick test (grade): Scoring of skin test results according to the guidance of American Academy of Allergy, Asthma and Immunology (AAAAI) and the American College of Allergy, Asthma and Immunology (ACAAI). The reference is “[Bernstein IL](http://www.ncbi.nlm.nih.gov/pubmed?term=Bernstein IL%5BAuthor%5D&cauthor=true&cauthor_uid=18431959), [Li JT](http://www.ncbi.nlm.nih.gov/pubmed?term=Li JT%5BAuthor%5D&cauthor=true&cauthor_uid=18431959), [Bernstein DI](http://www.ncbi.nlm.nih.gov/pubmed?term=Bernstein DI%5BAuthor%5D&cauthor=true&cauthor_uid=18431959), [Hamilton R](http://www.ncbi.nlm.nih.gov/pubmed?term=Hamilton R%5BAuthor%5D&cauthor=true&cauthor_uid=18431959), [Spector SL](http://www.ncbi.nlm.nih.gov/pubmed?term=Spector SL%5BAuthor%5D&cauthor=true&cauthor_uid=18431959), et al. (2008) Allergy diagnostic testing: an updated practice parameter. [Ann Allergy Asthma Immunol.](http://www.ncbi.nlm.nih.gov/pubmed/18431959#%23) 100(3 Suppl 3):S1-148.“
