# Supplementary material for: Alpha-Actinin Is a New Type of House Dust Mite Allergen
Source: PLoS One. 2013 Dec 6;8(12):e81377. doi: 10.1371/journal.pone.0081377 (PMC3855699; doi:10.1371/journal.pone.0081377)
Supplement: Table S2 — D. farinae allergens with molecular weight around 90 kDa identified by 2-DE and ESI-QUAD-TOF mass spectrometry. (DOCX) [file pone.0081377.s003.docx]

Table 2 *D. farinae* allergens with molecular weight around 90 kDa identified by 2-DE and ESI-QUAD-TOF mass spectrometry

Spot No. Name Molecule MW(kDa) pI Species Identical peptide span Mascot score (Fig. 2A & B, Fig. S1A-D)

1 Der f 15 Chitinase 95 6.18 *D. farinae* 126~136 171~181 273~284 275~284 294~301 397~406 432

2 Der f 15 Chitinase 95 5.3 *D. farinae*  48~70 126~136 273~291 275~284 285~291 397~406 510

3 Der f 15 Chitinase 95 4.67 *D. farinae*  24~34 48~60 126~136 171~181 275~284 285~291 468

4 Der f 24 Alpha-actinin 90 6.8 *Camponotus floridanus* 455~461 681~690 703~714 718~728 827~839 298
